# Supplementary material for: Matrix Metalloproteinase-9 Enhances Osteoclastogenesis: Insights from Transgenic Rabbit Bone Marrow Models and In Vitro Studies
Source: Int J Mol Sci. 2025 Mar 29;26(7):3194. doi: 10.3390/ijms26073194 (PMC11989254; doi:10.3390/ijms26073194)
Supplement: Supplementary file 1 [file ijms-26-03194-s001.zip › ijms-3501470-supplementary.pdf]

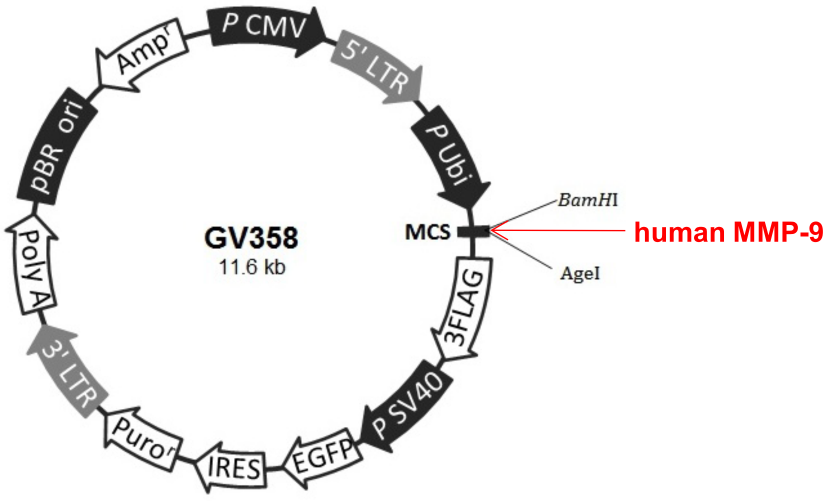

RAW264.7 cell stably expressed human MMP-9

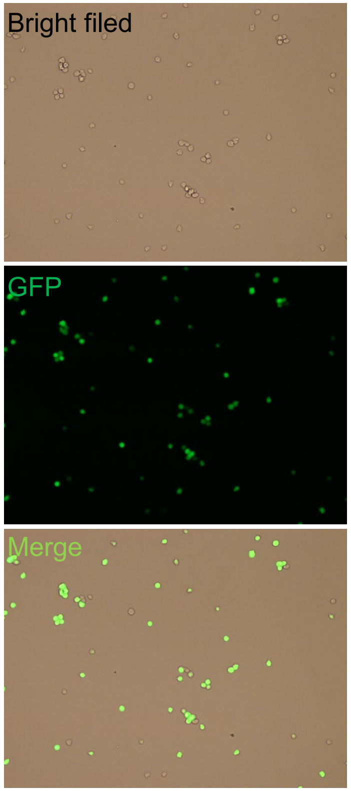

Lentiviral vector
Vector information :
General: 11615 bp
HIV-1_5_LTR,truncHIV-1_3_LTR: 835-1015
Ubiquitin Promoter: 2617-3833
3FLAG: 3880-3957
SV40 promoter: 3964-4341
EGFP: 4350-5069
IRES: 5092-5676
Puromycin: 5683-6282
HIV-1_5_LTR,truncHIV-1_3_LTR: 7420-7600
pBR322_origin: 10467-9848
Ampicillin: 11482-10622
CMV_immearly_promoter: 239-810

Supplement figure-S1
